# Current research progress of local drug delivery systems based on biodegradable polymers in treating chronic osteomyelitis

**DOI:** 10.3389/fbioe.2022.1042128

**Published:** 2022-11-24

**Authors:** Yixiu Liu, Xu Li, A. Liang

**Affiliations:** ^1^ Department of Orthopaedics, The Central Hospital Affiliated to Shenyang Medical College, Shenyang, China; ^2^ Shenyang Clinical Research Center for Hand and Foot, Shenyang, China

**Keywords:** chronic osteomyelitis, local drug delivery systems, synthetic biodegradable polymers, natural biodegradable polymers, micro/nanoparticles

## Abstract

Chronic osteomyelitis is one of the most challenging diseases in orthopedic treatment. It is usually treated with intravenous antibiotics and debridement in clinical practice, which also brings systemic drug side effects and bone defects. The local drug delivery system of antibiotics has the characteristics of targeted slow release to the lesion site, replacing systemic antibiotics and reducing the toxic and side effects of drugs. It can also increase the local drug concentration, achieve sound bacteriostatic effects, and promote bone healing and formation. Currently, PMMA beads are used in treating chronic osteomyelitis at home and abroad, but the chain beads need to be removed after a second operation, inconveniences patients. Biodegradable materials have been extensively studied as optimal options for antibiotic encapsulation and delivery, bringing new hope for treating chronic osteomyelitis. This article reviews the research progress of local drug delivery systems based on biodegradable polymers, including natural and synthetic ones, in treating chronic osteomyelitis.

## 1 Introduction

Chronic osteomyelitis is a severe infectious disease in bone tissue that easily occurs in vertebrae, feet of diabetes patients, and penetrating bone injury caused by trauma or surgery. It is usually caused by aerobic or anaerobic bacteria, mycobacterium, and fungi. After entering the human body, most bacteria adhere to the surface of necrotic soft tissue and bone tissue and form a biofilm that protects bacteria, significantly enhancing bacterial resistance and making it difficult for bacteria to be removed entirely. Therefore, chronic osteomyelitis is difficult to cure and becomes a major challenge for orthopedic surgery (Lew and Waldvogel, 2004). The incidence of chronic osteomyelitis has increased significantly with improved diagnosis and an aging population (Pollard et al., 2006; Trampuz and Zimmerli, 2006). The cost of treating implant-related chronic osteomyelitis in US hospitals is estimated to reach 1.62 billion US dollars by 2020 (Kurtz et al., 2012), bringing a heavy economic burden to patients and society. In addition, infection in ulcers of the chronic diabetic foot may lead to diabetic foot osteomyelitis, increasing mortality and amputation risk (Demetriou et al., 2013; Panagopoulos et al., 2015), which has been challenging for physicians and patients due to the limitation of recurrent and persistent infections.

The primary treatment of chronic osteomyelitis is complete debridement followed by long-term antimicrobial therapy (Walter et al., 2012; Zimmerli and Sendi, 2017). Debridement is a functional and practical approach to eradicating the infection in complicated chronic osteomyelitis (Inzana et al., 2016); it can reduce most bacteria in the infected area and optimize the soft tissues and vascular function in the surroundings. It is well known that systemic and local delivery of antibiotics could eradicate remaining bacteria. However, voids created during the deep debridement in bone tissue require bone grafts to fill, possibly causing further infection (Zhou et al., 2018a). In addition, long-term systemic administration of antibiotics is expensive and often causes systemic adverse effects such as nephrotoxicity and gastrointestinal discomfort (Huang et al., 2019). Oral or intravenous antibiotic therapy under ischemia is difficult to achieve adequate local antibiotic concentrations due to vascular damage to the infected bone; limited biofilm penetration makes managing chronic osteomyelitis more complex (Cobb et al., 2020). Therefore, antibiotic therapy focuses on utilizing topical delivery systems to achieve high concentrations of antibiotics at the infection site while avoiding side effects from systemic administration (Zhang et al., 2010), which plays an essential role in symptom relief and treating chronic osteomyelitis (Ahluwalia et al., 2021).

The application of bone cement loaded with antibiotics has been demonstrated as an efficient and commonly used strategy for treating infectious osteomyelitis due to the following advantages: 1) effective infection suppression in the early stage, 2) noticeable reduction in the incidence of recurrent infection, 3) decreasing the occurrence of pathological fracture caused by internal reinforcement, 4) suitable for bone regeneration (Dzyuba et al., 2016; Mauffrey et al., 2016; Wang et al., 2021). The most widely used bone cement material is poly(methyl methacrylate) (PMMA) beads with mixed antibiotics before surgery (Gogia et al., 2009), it offers high concentrations of antibiotics at the lesion site without causing hypersensitivity reactions (Lalidou et al., 2014) and is the gold standard of local delivery therapy for osteomyelitis (Mohanty et al., 2003). However, PMMA is non-biodegradable and requires removal *via* a second surgery (Mills et al., 2018); the stability of mixed antibiotics would be affected by the heat released during the preparation of PMMA beads (Nandi et al., 2009). Therefore, significant efforts have been devoted to developing biodegradable materials to prepare the local antibiotic delivery system for treating osteomyelitis (Nandi et al., 2009; Wassif et al., 2021).

## 2 Biodegradable polymers

As an essential branch of biodegradable materials, biodegradable polymers, including natural and synthetic polymers (Englert et al., 2018; Samadian et al., 2020; Guo et al., 2021; Jung et al., 2022), are of great importance in drug delivery and tissue engineering due to their excellent biocompatibility or low toxicity and biodegradation. Natural biodegradable polymers (collagen, chitosan, and silk protein), and synthetic polymers, such as poly-lactic acid (PLA), poly(lactic-co-glycolic) acid (PLGA), poly(ε-caprolactone) (PCL), and poly(trimethylene carbonate) (PTMC), offer great potential in designing delivery systems for local delivery of antimicrobial agents to the infected sites.

### 2.1 Natural biodegradable polymers

Natural polymers from nature are often used in biomedical applications because of their biodegradability, high biocompatibility, and low non-toxicity (Bhatia, 2016; Kaur et al., 2018; George et al., 2019). Among them, collagen, chitosan, and silk protein appear as sensible biomaterials for treating osteomyelitis (Dorati et al., 2017; Zhang et al., 2021; Zhang et al., 2022) due to their ability to promote cell adhesion and growth (O’brien, 2011).

#### 2.1.1 Collagen

Collagen, a natural protein in the extracellular matrix of bone, has become an ideal biomaterial for scaffolding material due to its high biocompatibility (Meyer, 2019). The antibiotics-loaded collagen has been extensively investigated in treating osteomyelitis, including acute and chronic ones. No removal of the resulting systems was required compared to PMMA (Atan et al., 2018) due to the excellent biodegradation behaviors.

Promising progress has been made in using collagen as an antibiotic delivery matrix to treat osteomyelitis. The commercially available antibiotic-loaded collagen sponge products have been developed (Table 1). The collagen matrix is processed into a sponge-like shape to increase the rate of collagen degradation and the level of antibiotic release, resulting in a better therapeutic effect. Some researchers have investigated the efficacy, evidence quality, and the *in vivo* pharmacokinetics of commercially available antibiotic-loaded collagen sponges in the clinical management of chronic osteomyelitis (Van Vugt et al., 2018). The results showed inadequate evidence quality and level of the included studies and high bias risk in these studies, making it challenging to guide any clinical decision. Hence, more convincing evidence is required for applying antibiotic-loaded collagen sponges in treating chronic osteomyelitis.

**TABLE 1:** Commercially available antibiotic-loaded collagen sponges.

Brand name	Antibiotics	Concentration (mg per 1 × 1 × 0.5 cm)	Collagen; origin
Septocoll	Gentamicin-sulfate/gentamicin-crobefate	1.2 mg gentamicin, 1.5 mg sulfate, 4.4 mg crobefate	Type I; equine
Sulmycin	Gentamicin-sulfate	1.43 mg gentamicin, 2.0 mg sulfate	Type I; equine or bovine

**FIGURE 1:**
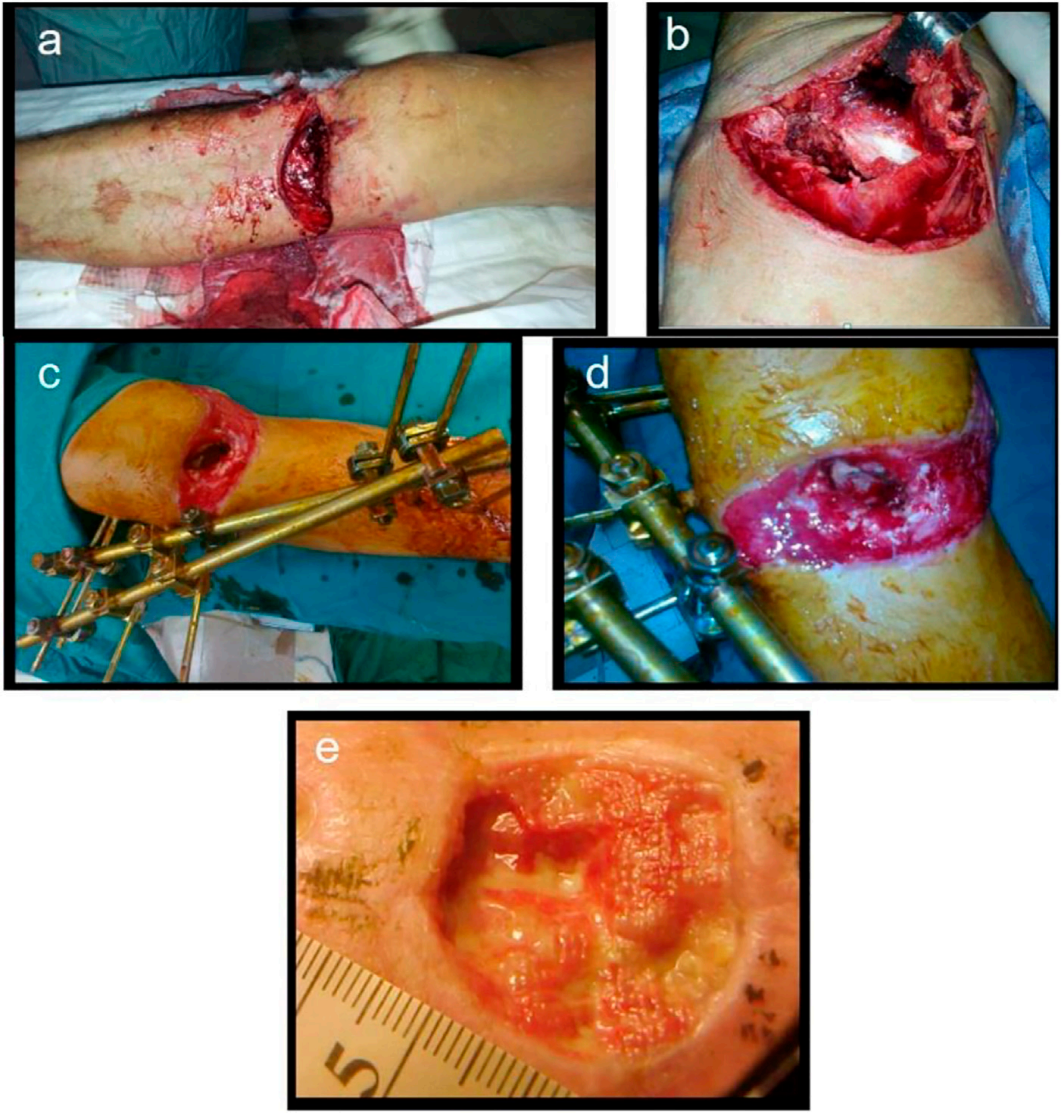
Open fracture of the proximal tibial metaphysis **(A)**, massive soft tissue injuries, periosteal stripping **(B)**, external fixator with knee spanning, bone defect visible **(C)**, Collatamp G filling the bone defect **(D)**, remaining skin defect, secondary epitelisation **(E)**. Reproduced with permission from Lupescu et al. (2016). Copyright Trans Tech Publications Ltd.

In addition, more mature delivery systems include gentamicin-containing collagen implants (GCCI), which have also progressed in preventing bone infections, and clinical research results have been reported one after another. Zawadzki et al. (2017) evaluated the efficacy of the GCCI in treating 103 patients with craniofacial and osteomyelitis, 54 patients received GCCI intraoperatively, and 49 were treated according to standard procedures as a control group. The study found that the course of postoperative antibiotic treatment and hospitalization was shorter, and the incidence of local complications was lower in GCCI patients, indicating a promotion for applying GCCI in treating osteomyelitis. In addition, Collatamp G, as one kind of GCCI, is a collagen-based sponge consisting of 280 mg collagen and 200 mg gentamicin, showing high treatment efficiency in post-traumatic bone infections (Deshmukh et al., 2016). Lupescu et al. (2016) described the clinical results after using Collatamp G, and the results are shown in Figure 1. The positive outcome for bone healing and infection control suggests that Collatamp G is a biomaterial that can address the abovementioned issues in treating bone infections.

**FIGURE 2:**
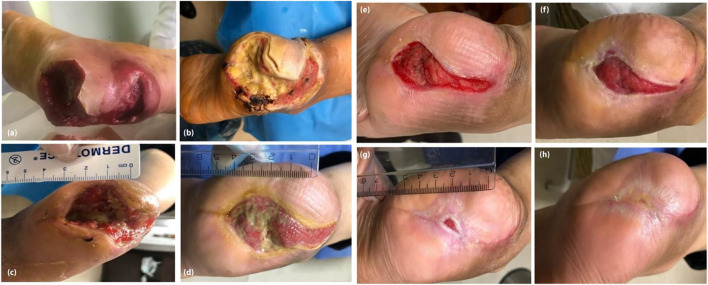
The patient before treatment **(A)**, 1 month **(B)**, 2 months **(C)**, 3 months **(D)**, 4 months **(E)**, 5 months **(F)**, 6 months **(G)**, and 7 months **(H)** after treatment. Reproduced with permission from Nilforoushzadeh et al. (2021). Copyright ^©^ 2020 The Authors.

A practical clinical case recently reported by Nilforoushzadeh et al. (2021). A type 2 diabetic patient with diabetic foot ulcer (DFU)-associated osteomyelitis were treated with a combination therapy of trichloroacetic acid, calcium alginate and foam dressings, human autologous fibroblast injection, and a fibroblast cell-seeded collagen scaffold. After treatment, the wound area was reduced by 90% (Figure 2), showing that the combination therapy positively affected DFU-induced osteomyelitis and could significantly reduce the risk of amputation in DFU patients. Although combination therapy is effective on DFU, some limitations must be solved: 1) the high cost of this combination therapy leads to difficulty in large-scale clinical promotion 2) an excellent cell bank is required on a large scale. 3) The preparation of the dressing was also tricky.

Recently, a new strategy has been developed to treat osteomyelitis by combining topical antibiotic delivery with a heparinized nano-hydroxyapatite/collagen bone substitute (Padrão et al., 2021). This work prepared particles of heparinized nano-hydroxyapatite/collagen biocomposites to load with vancomycin for treating osteomyelitis. After administration, the infection would be eradicated by the high concentrations of vancomycin provided by this system, and bone regeneration would be induced due to the regenerative scaffold role of the particles after antibiotic release. The study showed that the nano-hydroxyapatite/collagen particles could release high concentrations of vancomycin for 19 days above the MIC, which could completely inhibit the growth of MRSA and thus did not produce biofilm formation. Adjusting the sintering temperature enables the material to have a larger actual surface area with more binding sites, thus increasing vancomycin adsorption and further release. Moreover, the nano-hydroxyapatite/collagen biocomposites have good biocompatibility and no cytotoxic effect. Considering these results, the vancomycin-loaded nano-hydroxyapatite/collagen biocomposite was shown to be sufficient to resist bone infection and create a suitable environment for forming new bone in the defect area, offering a promising solution for the treatment of osteomyelitis. Sheehy et al. (2018) also explored the anti-infective effect of the collagen/hydroxyapatite antibiotic delivery system, using the commercially available antibiotic-eluting fleece Septocoll as the control group to evaluate the therapeutic effect on an animal model of chronic osteomyelitis. After 8 weeks of treatment, most of the rabbits in the blank group were still infected, while the infection rate was lower in both the antibiotic-eluting stent and the control groups. The results demonstrate that implantation of an antibiotic-loaded collagen-based material after debridement could enhance the bacterial clearance at the lesion site and improves the therapeutic effect of chronic osteomyelitis.

#### 2.1.2 Chitosan

Chitosan (CS) is a natural polymer from chitin. In addition to its biological properties (Li et al., 2020; Wang et al., 2020; Aranaz et al., 2021), such as biodegradability, biocompatibility, tissue engineering ability, and antibacterial activity, it also possesses chemical properties. It has been widely studied as a topical drug delivery vehicle for treating osteomyelitis *in vitro* and animal models (Wells et al., 2018; Sarwar et al., 2020).

The drug delivery systems based on CS present essential potential in treating infectious injuries. (Boles et al., 2018). The CS-polycaprolactone blend sponge can also be prepared to treat chronic osteomyelitis as a drug delivery system. The composite sponge can simultaneously load and adjust ciprofloxacin hydrochloride and ibuprofen release behavior, presenting a dual antimicrobial and anti-inflammatory activity to enhance the treatment therapy of chronic osteomyelitis. (Wei et al., 2019). The treatment effect of calcium sulfate-based drug delivery systems for chronic osteomyelitis could be improved by CS coating, which can affect the cell affinity and antibiotic elution *via* its deacetylation degree. Therefore, CS/calcium sulfate composite can release high concentrations of antibiotics and promote osteoblast adhesion, proliferation, and bone mineralization (Beenken et al., 2014).

**FIGURE 3:**
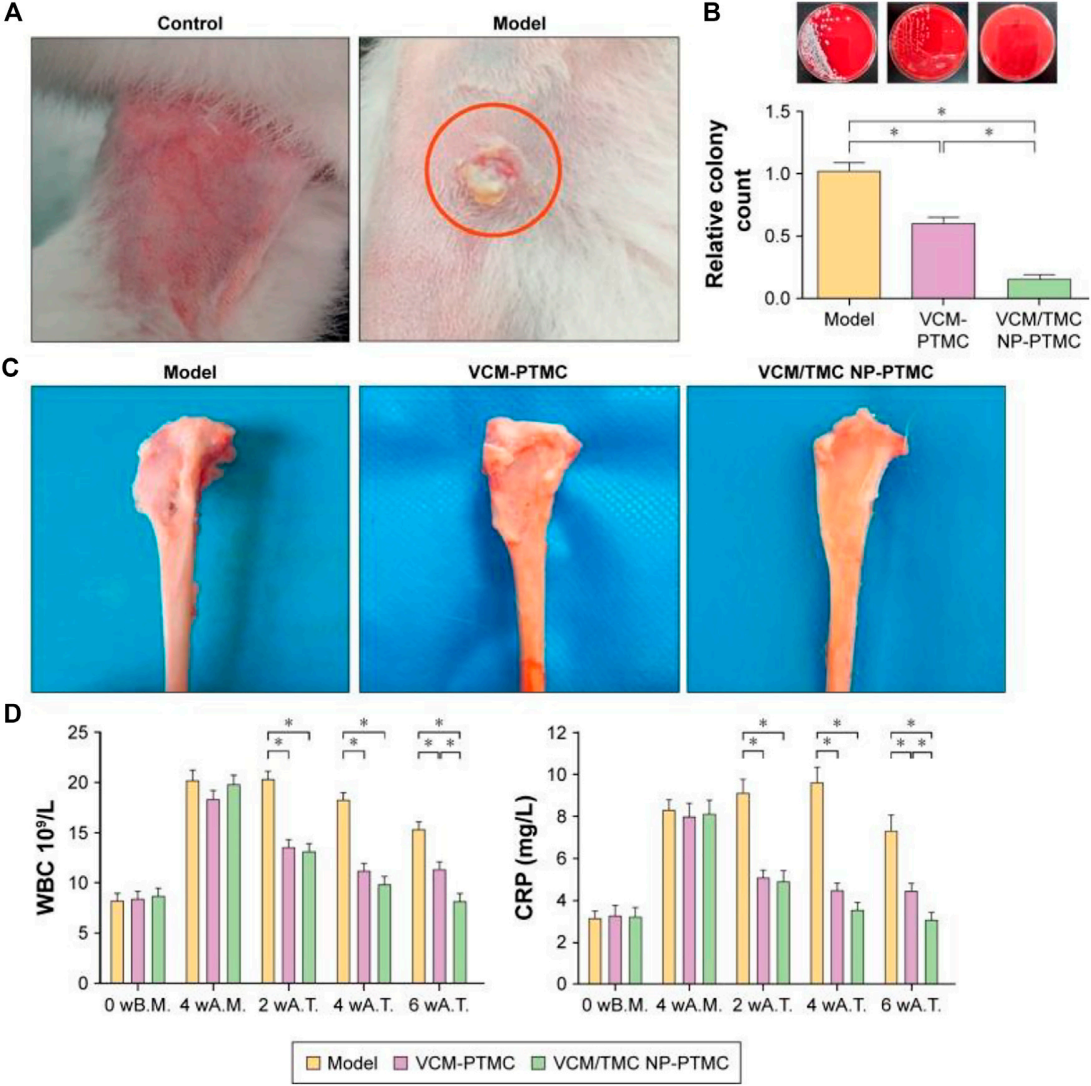
The antibacterial activity of VCM-PTMC and VCM/TMC-loaded PTMC nanoparticles. **(A)** The appearance of the rabbit legs at the fourth week after infection with *Staphylococcus aureus*. **(B)** A typical photograph of bacterial colonies forming on sheep blood agar plates and the number of bacterial colonies in the tibia marrow counted following overnight incubation. **(C)** Typical photographs of the tibia underwent 8 weeks of treatment with VCM-PTMC and VCM/TMC-loaded PTMC. **(D)** The results of WBC and CRP estimations in the rabbit serum at the time before modeling, fourth week after infection, fourth week after treatment, and eighth week after treatment. *p < 0.05. Reproduced with permission from Zhang et al. (2017). Copyright ^©^ 2017 The Authors.

In addition, some studies on chitosan and its derivative-based delivery systems have made some progress in repairing bone defects and promoting bone healing. These delivery systems can potentially be used in treating osteomyelitis when supplemented with antibiotic delivery. It has been reported that CS derivatives have unique biological properties that can improve the therapeutic effect of osteomyelitis and promote bone regeneration. CS derivatives have been found to have excellent efficiency in drug delivery for osteomyelitis treatment, as reported in various studies (Tao et al., 2021). N-trimethyl chitosan (TMC) is a derivative of CS, which is water-soluble and can be used to prepare highly efficient delivery systems of antibiotics. Zhang et al. (2017) reported that vancomycin-loaded N-trimethyl chitosan nanoparticles exhibited good biodegradability, cytocompatibility, and antibacterial properties. Vancomycin (VCM) showed smooth and sustained release kinetics, and no initial burst was observed, which benefited from polytrimethylene carbonate (PTMC) in the nano-drug delivery system. The surface erosion degradation mechanism of PTMC provided a sound sustained-release barrier to achieve long-acting sustained-release of antibiotics. In this drug delivery system, the active regulatory proteins, which are primarily adsorbed on the scaffold by positive charges, can promote the adhesion and proliferation of osteoblasts (Figure 3). The above advantages are crucial for promoting bone healing and repair, making this system a promising candidate for treating chronic osteomyelitis.

**FIGURE 4:**
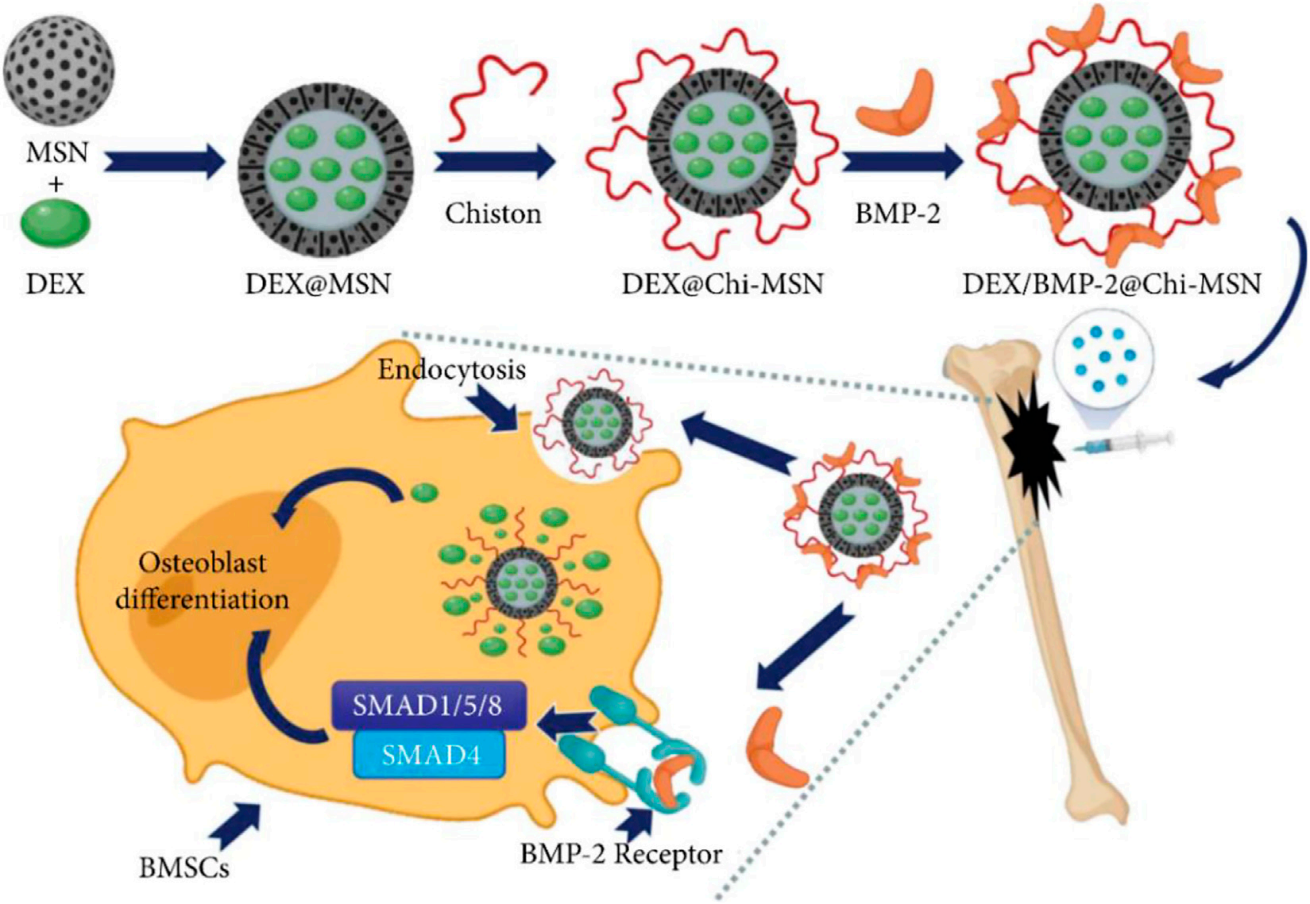
Mesoporous Silica Nanoparticle-Chitosan-Loaded BMP-2 in the Repair of Bone Defect in Chronic Osteomyelitis. Reproduced with permission from Yi et al. (2022). Copyright ^©^ 2022 The Authors.

The release and delivery of bone morphogenetic protein 2 (BMP-2) are critical for improving the clinical efficacy of bone healing and repair. To transfer BMP-2 to the target area, Yi et al. (2022) constructed a novel nano-delivery system (Chi-MSN) composed of mesoporous silica nanoparticles (MSN) and chitosan. The study reported that the Chi-MSN system could effectively reduce drug loss and deliver BMP-2 to the lesion site due to its stable and pH-responsive properties. In addition, Chi-MSN can better penetrate cells, thus better enhancing cell viability and reducing apoptosis. In the *in vivo* experiments, the defect in bone tissue was better repaired in the Chi-MSN group, as indicated by the increased number and thickness of trabecular bone in this group (Figure 4). Therefore, the Chi-MSN delivery system may be a promising candidate for future bone repair in chronic osteomyelitis.

The lack of effective delivery methods limits the high-concentration release of VCM in irregular bone tissue, resulting in suboptimal infection therapy. Recently, chitosan-based thermosensitive hydrogel loading VCM nanoparticles (VCM-NPs/Gel) have been designed and prepared to simultaneously prevent infection and repair fractures, showing activity against *Staphylococcus aureus* due to the sustained release of VCM for more than 26 days, promoting osteoblast proliferation. Furthermore, the release mechanism of VCM-NPs/Gel was the diffusion and degradation of the hydrogel matrix, which together maintained the stable release of VCM. The results *in vivo* showed that the investigated system had prominent anti-infective properties and accelerated bone repair and regeneration during osteomyelitis treatment, showing great potential as an effective strategy for treating osteomyelitis (Tao et al., 2020).

#### 2.1.3 Silk protein

Like chitosan and collagen, silk protein such as that produced by silkworms and spiders also has a comprehensive source. It also has more robust mechanical properties, excellent biocompatibility, and low immunogenic response (Zapata et al., 2022). Furthermore, it can be processed into various structures, such as hydrogels, fibers, membranes, microspheres, and nanospheres (Altman et al., 2003; Koh et al., 2015; Mottaghitalab et al., 2015), making it more suitable for orthopedic applications. For example, the vancomycin-loaded silk nanospheres could effectively clear the bacteria from the infection site (Hassani Besheli et al., 2017; Mulinti et al., 2021). Silk protein incorporated with HA has also been demonstrated to be a strategy for cell proliferation and adhesion to enhance the growth of bone (Saleem et al., 2020).

### 2.2 Synthetic biodegradable polymers

The properties of synthetic biodegradable polymers can be precisely tailored to the needs of the application, including physical and mechanical properties, as well as biodegradability, which facilitates adjustment of the release rate of therapeutic agents such as antibiotics for better therapeutic effects. In addition, the performance of different synthetic batches of polymers is also more stable and reliable, suitable for mass production and clinical applications. Among the synthetic biodegradable polymers, polylactic acid (PLA), poly(lactic-co-glycolic acid) acid (PLGA), poly(ε-caprolactone) (PCL), and poly(trimethylene carbonate) (PTMC), and drug delivery systems constructed with them as carrier materials play an essential role in the treatment of chronic osteomyelitis.

#### 2.2.1 Polylactic acid

Polylactic acid (PLA) has good biocompatibility, biodegradability, a wide range of mechanical and physical properties, and low immunogenicity and has been approved by the Food and Drug Administration. Therefore, PLA has been the focus of numerous preclinical and clinical trials, especially in drug delivery and bone tissue engineering (Tyler et al., 2016; Liu et al., 2020).

**FIGURE 5:**
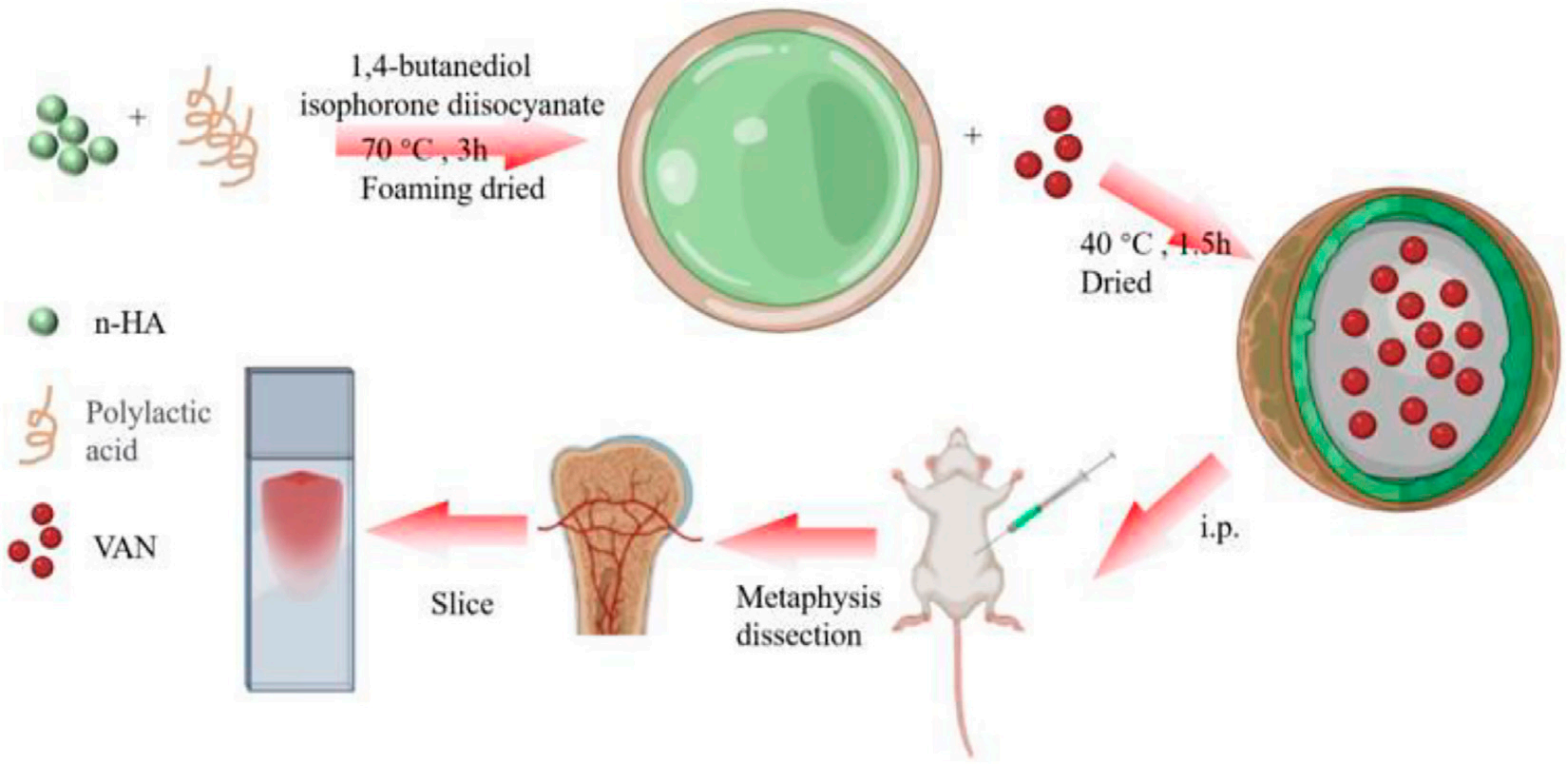
nHA-PLA-VAN system assembling and study strategy. Reproduced with permission from Lv et al. (2022). Copyright ^©^ 2022 The Authors.

The acidic products generated in the degradation process of PLA can easily induce an inflammatory response. To overcome the problems caused by acidic degradation products, PLA is usually combined with hydroxyapatite (HA) to form a composite material. Lv et al. (2022) prepared a nanodevice based on nHA-PLA to deliver vancomycin (VAN) in treating chronic osteomyelitis (Figure 5). This study found that nHA-PLA showed good biocompatibility and degradability. It could effectively release VAN into the lesion and deliver it to the bone marrow tissue, thereby better inhibiting bacteria and inflammatory reactions. Meanwhile, the sound osteoconductive and osteoinductive effects of nHA-PLA-VAN help promote osteoblasts’ adhesion and proliferation, thereby achieving a better repair of bone defects. In a model of chronic osteomyelitis, nHA-PLA-VAN was found to be effective in reducing inflammatory reactions, promoting the construction of medullary cancellous bone, and helping restore the biomechanical properties of bone. Therefore, nHA-PLA nanodevice loading vancomycin has great potential in treating chronic osteomyelitis. A similar observation was also found in the work of Zhao (Zhao et al., 2019). They developed local drug delivery beads of ofloxacin, consisting of poly(sebacic anhydride) (PSA) and poly-D, L-lactide (PDLLA), for treating chronic osteomyelitis. The delivery system with 90 wt% PDLLA produced a prominent inhibition effect against bacteria *Staphylococcus aureus*, *Escherichia coli*, and *Pseudomonas aeruginosa* within 89 days. Drug release in the local bone of rabbits showed that the mean concentration of ofloxacin was 20.1 ± 10.3 μg/g over 8 weeks, and the mean concentration in plasma was 35.6 ± 18.8 ng/ml. Radiography, bacterial culture, and histology showed an excellent therapy of osteomyelitis in rabbits, suggesting that PSA/PLA mixtures as antibiotic carriers may help treat chronic osteomyelitis and prevent bone infections (Chen et al., 2007).

Recently, PLA and PCL (PLC) copolymers have been investigated for bone repair and drug delivery (Sang et al., 2018; Mendibil et al., 2019). Biodegradable scaffolds of PLC composited with calcium phosphate (CaP) were prepared for delivery of the antibiotic moxifloxacin hydrochloride (MOX) (Radwan et al., 2021). The resulting scaffolds showed sustained release of MOX for 6 weeks and maintained cell proliferation and differentiation, thereby reducing inflammation and sequestrum formation in the bones caused by chronic osteomyelitis. The results indicate that PLC/CaP scaffolds are favorable candidates for chronic osteomyelitis therapy and suggest further clinical trials.

#### 2.2.2 Poly-(lactic-co-glycolic acid)

Poly-(lactic-co-glycolic acid) (PLGA) is the copolymer of lactic acid (LA) and glycolic acid (GA), which is one of the most accepted materials for controlled drug delivery and bone tissue engineering (Lagreca et al., 2020; Jin et al., 2021) and has been approved by Food and Drug Administration (FDA) in the clinic because of its excellent biodegradability and biocompatibility.

Currently, treating chronic osteomyelitis presents a significant challenge to clinical orthopedics. Combining PLGA and antibiotics provides a new option for treating chronic osteomyelitis. PLGA microspheres can improve the encapsulation rate of antibiotics, alleviate the initial burst release, reduce the cumulative release of drugs, prolong the action time of drugs (Yang et al., 2017; Li et al., 2022), and improve the antibacterial activity *in vitro* and *in vivo* (Cevher et al., 2007; Posadowska et al., 2015).

Novel inorganic-organic composites are potential materials for treating chronic osteomyelitis or infected bone defects. Mistry et al. (2022) reported the efficacy of antibiotic-loaded PLGA/biphasic calcium phosphate composite bone cement in treating experimental osteomyelitis. Compared with PMMA cement, PLGA composite cement showed superior cytocompatibility and coagulation activity, enabling faster and better sepsis control and promoting bone regeneration, indicating that PLGA cement is a promising carrier of the antibiotic-loaded delivery system for treating chronic osteomyelitis without the removal of cement. Cheng’s group prepared vancomycin-loaded bioactive glass (MBG)/PLGA scaffolds for bone tissue engineering (Cheng et al., 2018). Compared with pure PLGA scaffolds, MBG/PLGA scaffolds exhibited better cytocompatibility and osteoblast differentiation properties. Vancomycin-loaded MBG/PLGA scaffolds exhibited good release properties and biocompatibility, which can sustainably release vancomycin for more than 8 weeks *in vitro*, inhibiting biofilm formation without adversely affecting cells. Gatifloxacin-loaded PLGA and β-tricalcium phosphate composite also demonstrated sufficient *in vitro* bactericidal activity and could significantly reduce inflammation within the debridement area, accompanied by osteoconduction and vascularization (Tamazawa et al., 2011; Kimishima et al., 2016). Likewise, the composites of gatifloxacin (GLFX)-loaded PLGA and hydroxyapatite (HA) (Makiishi et al., 2017) could maintain sufficient bactericidal activities from 3 h to 10 days. After 4-week implantation in bone defects of osteomyelitis, the inflammation was significantly reduced (*p* < 0.05), and the formation of new bone can be found (Makiishi et al., 2017), as compared to the debridement group. These findings show that PLGA composites could control bacterial infection and support bone regeneration for osteomyelitis treatment.

In addition to the above, to avoid the side effects caused by systemic antibiotic therapy for treating osteomyelitis, a novel sol-gel drug delivery system was developed consisting of polyethylene glycol monomethyl ether (mPEG) and PLGA, which offered several advantages, such as easy preparation, high encapsulation efficiency, zero-order release, injectable and *in situ* gelling in lesion sits. The implantation of teicoplanin-containing mPEG-PLGA hydrogel is effective in treating rabbit osteomyelitis and may have great promise as a therapeutic strategy for chronic osteomyelitis (Peng et al., 2010).

#### 2.2.3 Poly(ε-caprolactone)

Poly(ε-caprolactone) (PCL) is a well-known biodegradable polymer with good biocompatibility. Research on PCL and its applications in drug delivery and other medical fields have recently received extensive attention.

While treating chronic osteomyelitis, insufficient antibiotics concentration at the infected lesions discounted the treatment therapy, so significant effort has been devoted to novel delivery systems to achieve sustained high concentrations without accompanying systemic side effects; for example, a rifampicin-loaded 3D-printed PCL scaffold was developed to treat osteomyelitis (Lee et al., 2020). The growth inhibitory activity against the representative pathogenic bacteria of osteomyelitis confirmed the excellent therapeutic effect. Maiti et al. (2018) developed a vancomycin-loaded PCL chip to treat MRSA-infected osteomyelitis, which can eliminate and recover bone, suggesting the efficacy of sustained release. The findings reported by Wei et al. (2018) also suggested that vancomycin-loaded PCL membranes have great potential in effectively controlling bone infection and promoting bone regeneration.

Blending can improve the properties of the parent materials and is an effective strategy to achieve desired target properties. PCL has the advantages of low toxicity, good mechanical strength, and controlled release properties. However, it lacks cellular recognition signals, while natural polymers possess cell-affinity sites, which can compensate for PCL’s lack of cell-affinity. Therefore, blending natural polymers and PCL will provide a better biomaterial for treating chronic osteomyelitis. Pawar and Srivastava (2019) prepared mixed sponges of PCL and chitosan to control ciprofloxacin hydrochloride and ibuprofen to treat chronic osteomyelitis, concluding that sponges are promising candidates for chronic osteomyelitis management after surgical debridement due to the ideal release profiles and potential antibacterial and anti-inflammatory activities. The 3D-printed PCL/alginate scaffolds containing antibiotics (Lee et al., 2022) were also demonstrated as a novel osteomyelitis treatment inhibiting biofilm formation and bacterial activity.

PLGC-based delivery systems loaded with ciprofloxacin are capable of maintaining sustained release of antibiotics for up to 30 days with sufficient concentrations to sustain long-term antimicrobial activity.

The PLGC-based and ciprofloxacin-loaded delivery system maintained sustained release of antibiotics for up to 30 days with sufficient concentrations to maintain long-term antibacterial activity.

**FIGURE 6:**
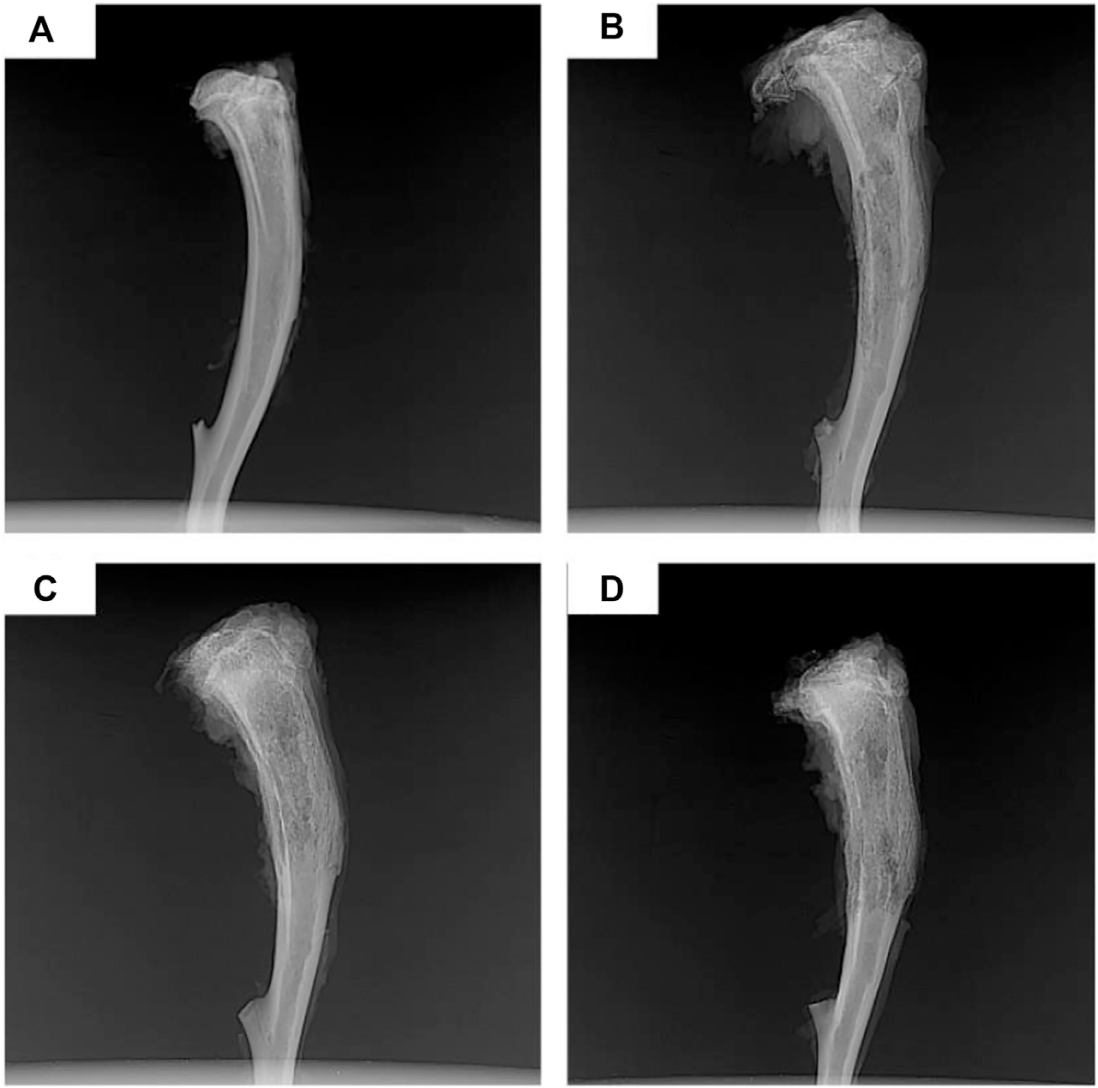
X-rays of chronic-osteomyelitis-model rats after 4 weeks of treatments: **(A)** Ciprofloxacin-PLGC system, **(B)** pure ciprofloxacin, **(C)** blank PLGC, and **(D)** no treatment. Reproduced with permission from Liu et al. (2020). Copyright ^©^ 2020 The Authors.

Although PLGA-based antibiotic delivery systems are promising candidates for treating chronic osteomyelitis, the higher glass-transition temperature hinders the implantation of matrix PLGA-based delivery systems into the bone marrow cavity. The introduction of PCL structure into the PLGA chain endows better flexibility to the resulting copolymer poly(d, l-lactide-co-glycolide-co-ε-caprolactone) (PLGC). PLGC-based delivery systems loaded with ciprofloxacin are capable of maintaining sustained release of antibiotics for up to 30 days with sufficient concentrations to sustain long-term antimicrobial activity (Liu and Bai, 2020). In a rat model of chronic osteomyelitis, the significant antibacterial effect of the PLGC/ciprofloxacin system was confirmed by the returned normal structure of proximal and middle tibiae (Figure 6), indicating that the PLGC-based local antibiotic delivery system is a suitable candidate for treating chronic osteomyelitis.

In a study, three-dimensional (3D)-printed antibiotic-loaded biodegradable scaffolds made of PCL/PLGA/tobramycin were reported for the first time for the treatment of chronic osteomyelitis (Shim et al., 2015), which was shown to be effective in antibacterial activity against *S. aureus* and *E. coli* and non-toxic to the proliferation of MG63 cells. The anti-inflammatory effect of PCL/PLGA/tobramycin scaffolds was further confirmed by gene expression (TNF-α, IL-6) in RAW 264.7 cells. In a rat model of chronic osteomyelitis, the tobramycin-loaded PCL/PLGA scaffold significantly reduced the infection-induced edema and inflammation. It promoted new bone formation after 8 weeks of implantation. The above findings suggest that 3D printed PCL/PLGA/tobramycin scaffolds can eradicate osteomyelitis and promote bone regeneration, showing great potential as local antibiotics delivery system in treating osteomyelitis. Moreover, the drug delivery systems are prepared from the composites of PCL with inorganics, such as calcium sulfate (Gupta et al., 2009; Yaprakci et al., 2013; Zhou et al., 2018b; Kyriacou et al., 2020) and calcium phosphate (Miyai et al., 2008; Kundu et al., 2012; Makarov et al., 2014; Kamboj et al., 2019) have modified drug release behavior that can continuously release sufficient concentrations of antibiotics and simultaneously promote bone regeneration, also demonstrating an efficient strategy in treating chronic osteomyelitis.

#### 2.2.4 Poly(trimethylene carbonate)

**FIGURE 7:**
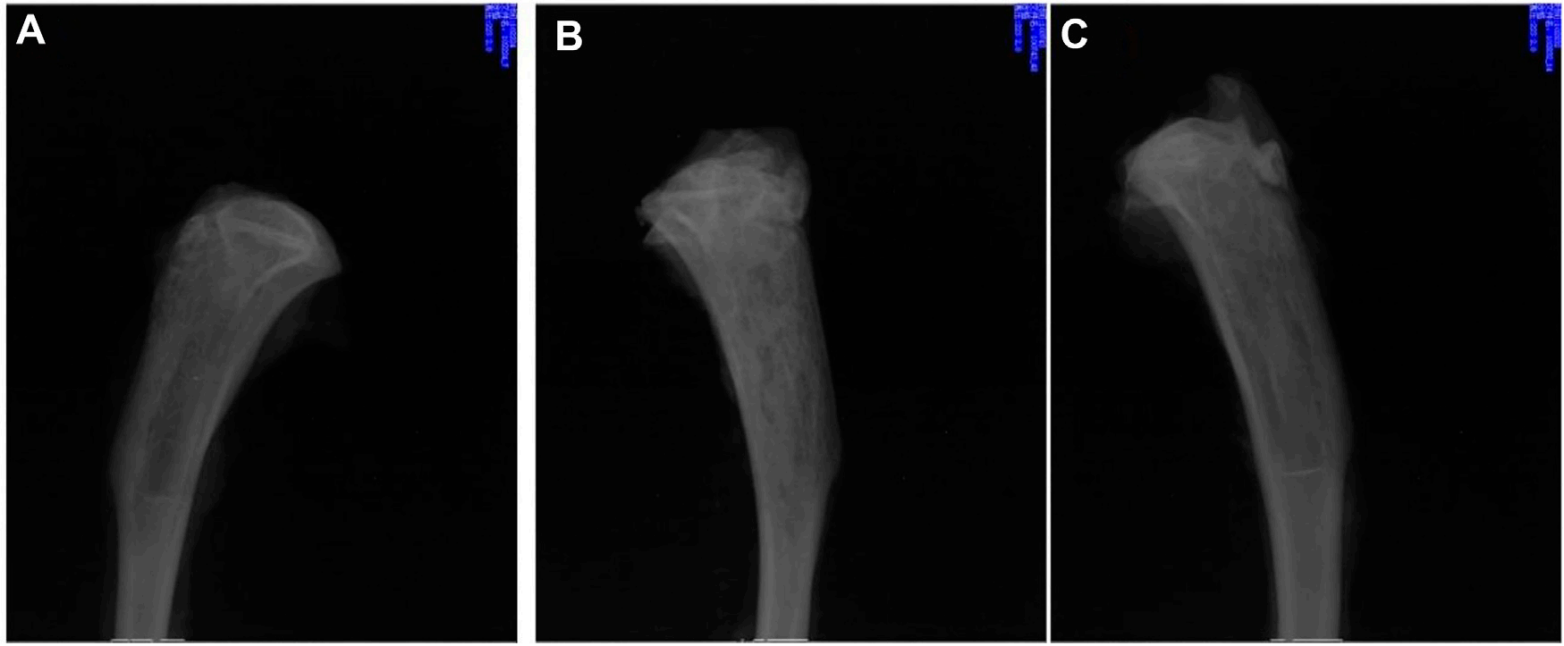
X-rays of chronic-osteomyelitis-mode. Treatment group (treated with ciprofloxacin-loaded PTMC implants) **(A)**, control group (treated with PTMC implants without ciprofloxacin) **(B)**, and blank group (no treatment) **(C)** after 28 days of treatments. Reproduced with permission from Liu et al. (2022). Copyright ^©^ 2022 The Authors.

The above reports have confirmed that aliphatic polyesters, such as PLA, PLGA, PCL and their copolymers, or composites formed by blending with inorganic materials have great potential as a matrix for delivery systems in the field of bone tissue repair. However, studies have shown that aliphatic polyesters produce acidic degradation products during the degradation process, which can lead to sterile inflammation, bacterial growth at bone lesions, and bone resorption or bone loss (Gunatillake et al., 2003; Böstman et al., 2005; Reich et al., 2020). Hence, further investigation of alternative materials without acidic degradation products is required. Poly(trimethylene carbonate) (PTMC) is a possible candidate material with good biocompatibility and controllable degradation rate without acidic degradation products (Yang et al., 2015; Yang et al., 2016; Hou et al., 2017; Hou et al., 2019; Wuyuntana et al., 2019; Hou et al., 2020; Cai et al., 2021; Hou et al., 2021; Hou et al., 2022), which may provide sustained high antibiotic release rates. As expected, gentamicin-loaded PTMC discs (Neut et al., 2009), ciprofloxacin-loaded PTMC implants (Liu et al., 2022), and vancomycin-loaded PTMC nanoparticles (Zhang et al., 2017) show characteristics of antibiotic-controlled release and biofilm inhibition (Figure 7). Hence, PTMC is also a promising potential carrier for local antibiotic delivery systems in treating osteomyelitis.

The synthetic polymers above include aliphatic polyesters and aliphatic polycarbonates, and their excellent biocompatibility and low toxicity have been confirmed in previous reports (Dubois et al., 1999; Suriano et al., 2011; Tempelaar et al., 2013; Mespouille et al., 2014; Ghosh et al., 2019). And their degradation products, after being degraded *in vivo* are non-toxic and can be absorbed by the body or excreted with metabolism (Brannigan and Dove, 2017; Xu et al., 2020). However, the degradation products of polyester materials can cause a decrease in the pH of the local microenvironment and are prone to cause sterile inflammation (Srivastava et al., 2020). This problem also needs to be considered and solved in practical applications.

## 3 Future perspectives

In summary, the local drug delivery system based on the degradable polymer has potential clinical applications in treating osteomyelitis. Despite this, much work is still desired in the properties of the biodegradable polymeric carriers, the kinetics of antibiotic release, and the further development of current systems. For example, in terms of the composition of the carrier, composite materials have more advantages, which can make up for the deficiencies of various materials and exert their respective advantages. Since about 65% nano-HA and 35% collagen in human bone tissue, researchers tend to use inorganic-organic combinations such as adding calcium phosphate to polymers and using Ca^2+^ to promote bone defect repair; In terms of material selection, since the acidic degradation products produced by polyester are harmful to local tissue growth, the reduction of local pH may also affect the biological activity of antibiotics, and drug delivery systems based on biodegradable polycarbonate may be more suitable for the chronic osteomyelitis treatment; The shape of local drug delivery systems is often composed of microspheres and nanoparticles, which can provide larger surface area to increase the drug loading capacity. In addition to ensuring sufficient effective concentration, another research focus is to match the degradation rate of biodegradable polymers with the growth rate of bone tissue to realize bone tissue regeneration. The local antibiotic delivery system based on biodegradable polymers is a promising strategy for treating osteomyelitis. Good results have been achieved in many animal models and a small number of clinical trials.

The future development direction will combine different biomaterials to complement their advantages and disadvantages. The developed new composite material carrier should have biodegradability, good biocompatibility, and low toxicity and be able to tune the release kinetics of antibiotics by tailoring the performance parameters of the carrier materials to achieve a long-lasting and stable release process. Furthermore, the focus should be placed on studying the pharmacokinetics and pharmacodynamics of novel antibiotic delivery systems *in vivo*. Moreover, fluorescence technology for antibiotic labeling could be attempted for visualization study. The final development direction is to reduce the cost of treatment of osteomyelitis, enhance treatment efficacy, and improve patient compliance.

## 4 Conclusion

This review discusses the research progress of biodegradable polymer-based drug delivery systems for treating chronic osteomyelitis. In conclusion, it is imperative to design new antibiotic-loaded local delivery systems with desirable properties for treating chronic osteomyelitis early and after debridement. The biodegradability, biocompatibility, and drug release properties of the polymer-carriers are essential to ensure that the drug delivery system can provide a microenvironment and mechanical support for the regeneration of new bone tissue during healing while at the same time avoiding long-term systemic therapy. Other biodegradable polymer-based antibiotic delivery systems are still in primary or preclinical research, except for collagen-based antibiotic delivery systems. There is a long distance to achieve actual practice in the clinic. The joint efforts of the multidisciplinary integration of biomedical polymer science, orthopedics, pharmacy, and clinical medicine are urgently required to accelerate this process.
